# Longitudinal and cross-sectional study of retinal phenotypes and visual function in choroideremia carriers: a new grading system

**DOI:** 10.1186/s40662-026-00476-2

**Published:** 2026-02-24

**Authors:** Xiaoxu Han, Yang Yu, Jiaqi Ding, Zixi Sun, Hui Li, Xuan Zou, Ruifang Sui

**Affiliations:** 1 Department of Ophthalmology, Peking Union Medical College Hospital, Chinese Academy of Medical Sciences and Peking Union Medical College, 1 Shuai Fu Yuan, Beijing, 100730 China; 2 Beijing Key Laboratory of Fundus Diseases Intelligent Diagnosis and Drug/Device Development and Translation, Beijing, 100730 China; 3 Department of Epidemiology and Biostatistics, Institute of Basic Medical Sciences, Chinese Academy of Medical Sciences and School of Basic Medicine, Peking Union Medical College, Beijing, 100005 China

**Keywords:** Choroideremia, Female carrier, Retinal phenotype, Visual function, Natural history

## Abstract

**Background:**

Choroideremia is an X-linked chorioretinal dystrophy with well-characterized progression in affected males but variable phenotypes in female carriers. Understanding the phenotypic spectrum in female carriers is important for prognosis, monitoring, and trial design. This study aims to delineate the natural history of retinal phenotypes and visual function loss in female choroideremia carriers and establish an improved fundus grading system for disease stratification and prognostic prediction.

**Methods:**

This single-center, longitudinal and cross-sectional, retrospective study included 64 genetically confirmed female choroideremia carriers. Clinical data included genotype, age, best-corrected visual acuity, color fundus photography, fundus autofluorescence, visual field testing, and full-field electroretinography. A novel fundus phenotypic grading system was proposed based on fundus autofluorescence and fundus color photographs, which included four types: granular (merged fine/coarse patterns), severe peripapillary atrophy (highlighting severe peripapillary atrophy as a crucial feature), localized atrophy, and widespread atrophy. The agreement between measurement-based grading and visual grading was assessed.

**Results:**

Visual acuity and fundus phenotypes showed moderate interocular symmetry, while visual field and electroretinography metrics showed high interocular symmetry. At baseline, phenotypes included granular (76.3%), severe peripapillary atrophy (7.5%), localized atrophy (10.8%), and widespread atrophy (5.4%). Longitudinally, the granular type remained stable, while other types progressed, with a mean atrophy expansion rate of 3.1 mm^2^/year. Age did not correlate with visual function decline, and neither age nor genotype was linked to the severe fundus phenotype. Baseline phenotype was the strongest predictor of prognosis. Excellent agreement (weighted κ = 0.93) was observed between the measurement-based and visual grading methods.

**Conclusions:**

We proposed a novel fundus grading system for choroideremia carriers and demonstrated its strong clinical utility and prognostic value. The granular type confers a favorable prognosis, whereas the other three types exhibit progressive deterioration. Baseline phenotypic grading is the best indicator of long-term outcomes, underscoring its value in clinical monitoring and therapeutic trial design.

## Background

Choroideremia (CHM) is an X-linked chorioretinal dystrophy characterized by progressive degeneration of the retinal pigment epithelium (RPE), photoreceptors, and choroid due to mutations in the *CHM* gene [1]. *CHM* encodes Rab escort protein-1, a geranylgeranyl transferase in the prenylation of Rab proteins and functions as a chaperone protein in intracellular vesicle trafficking [2]. The exact mechanism of retinal degeneration in CHM remains unclear, and debate continues over whether degeneration begins in the photoreceptors [3–5], the RPE [6–10], or whether both deteriorate simultaneously [11]. However, the broad consensus is that choroidal tissue loss occurs secondary to RPE and photoreceptor degeneration [4].

Affected males with CHM typically present with night blindness in childhood, followed by a gradual loss of peripheral vision, often resulting in tunnel vision and, ultimately, legal blindness [12]. Although female carriers have historically been considered asymptomatic, a recent study [13] using the Michigan Retinal Degeneration Questionnaire has revealed self-reported visual symptoms even among those with mild fundus phenotypes. A small number of female carriers experience symptoms similar to those of male patients.

Fundus manifestations in female carriers of CHM display substantial phenotypic variability, ranging from near-normal retinal appearance to severe chorioretinal degeneration. This phenotypic spectrum is mainly attributed to the mechanism of skewed X-chromosome inactivation, wherein the inactivation ratio of normal *CHM* allele directly correlates with the severity of fundus manifestations [14]. To date, two distinct grading systems have been published for characterizing fundus phenotypes in female carriers of CHM. Edwards et al. [15] first introduced a four-tier classification system, classifying phenotypes into fine, coarse, geographic, and male patterns. Subsequently, Jauregui et al. [16] proposed a modified three-tier grading system, categorizing disease severity as mild, intermediate, or severe. Notably, both grading systems were developed based on relatively small cohorts, with each study including 12 CHM carriers in their respective analyses.

**Fig. 1:**
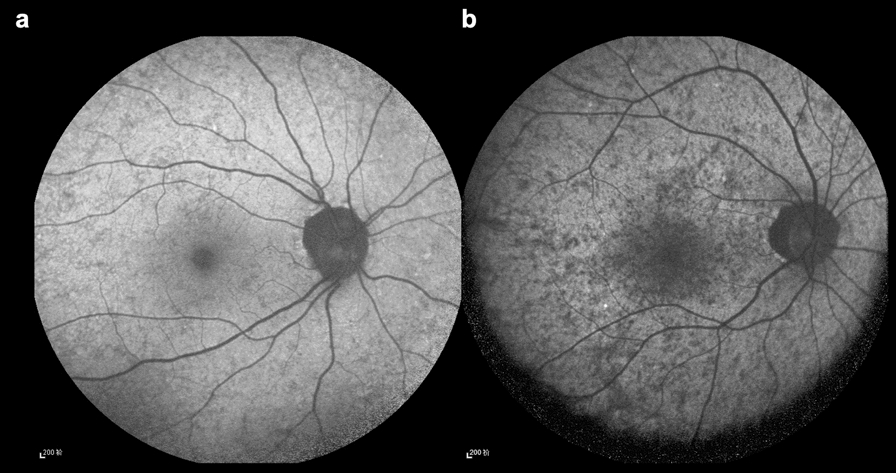
Illustration of fine and coarse patterns, according to the grading system established by Edwards et al. [15] using fundus autofluorescence (FAF) imaging. **a** Fine pattern; **b** Coarse pattern

In clinical practice, distinguishing between fine and coarse patterns remains challenging due to the lack of well-defined criteria in existing grading systems. Figure 1 illustrates our representation of the fine and coarse patterns, although accurate discrimination between these patterns often proves challenging. Furthermore, this differentiation appears to have limited clinical significance when evaluating disease severity and prognostic outcomes. Concurrently, we identified that severe peripapillary atrophy may represent a characteristic phenotype with potential value in disease assessment and prognosis. These observations prompted us to develop a revised grading system to better characterize disease manifestations and enhance clinical decision-making.

Recently, Gocuk et al. [13] published a cross-sectional study on the retinal characteristics of 43 CHM carriers from Australia and the United Kingdom based on multimodal imaging and a longitudinal study on the retinal characteristics of 17 CHM carriers [17]. These studies investigated the visual acuity (VA), optical coherence tomography (OCT), microperimetry, and fundus autofluorescence (FAF) of carriers, advancing our understanding of the disease. However, existing studies have not adequately described the characteristics of color fundus photography, visual field (VF) defects, and full-field electroretinogram (ffERG) abnormalities in large CHM carrier cohorts. Additionally, no relevant studies currently address the proportion of severe cases, prognostic differences among different fundus phenotypes, or factors associated with severe cases in other populations.

Here, our longitudinal and cross-sectional study aim to characterize fundus manifestations, visual function, and interocular symmetry in a large cohort of molecularly confirmed CHM carriers with various disease severities while exploring genotype–phenotype correlations. Furthermore, we propose a novel fundus phenotype grading system to better guide the grading of disease conditions, predict disease prognosis, and provide guidance for genetic therapy interventions.

## Methods

### Study participants

This single-center, retrospective cohort study enrolled heterozygous female carriers of *CHM* mutations. The diagnosis was based on presenting symptoms, family history, and fundus examination, and CHM carriers were identified through the relationship with the affected probands and subsequently screened for the heterozygous disease-causing variant. The study protocol was approved by the Institutional Review Board of Peking Union Medical College Hospital (No. K1322), and the study was conducted in accordance with the principles of the Declaration of Helsinki. Written informed consent was obtained from all participants.

### Clinical evaluation

All follow-up visits for CHM carriers were reviewed, with longitudinal data included only when the interval between visits was ≥ 1 year; shorter intervals were excluded. The systematically collected data included demographics, ocular history, medical/surgical history, age at visit, best-corrected visual acuity (BCVA), refractive error, slit-lamp and dilated fundoscopic examination findings, color fundus photography, short-wavelength FAF, VF, and ffERG. All participants were observed by the same inherited retinal dystrophy (IRD) specialists (RS and XH).

### Measurement and analysis for visual function and structure

Snellen VA test results were converted to decimal and logarithm of the minimum angle of resolution (logMAR) unit value using the following formula: logMAR =  − log (decimal acuity) for subsequent analysis. Participants’ abilities to count fingers, detect hand movements, and detect light perception were assigned logMAR values of 2.6, 2.7, and 2.8, respectively [18].

Color fundus photography (Topcon, Tokyo, Japan; Optos California, Optos GmbH, Germany) and FAF (Heidelberg Engineering, Heidelberg, Germany; Optos California, Optos GmbH, Germany) were recorded with standardized setups. The Heidelberg Eye Explorer software (Heidelberg Engineering, Heidelberg, Germany) was used to measure the area of retinal atrophy, the horizontal width of peripapillary atrophy, and the horizontal disc diameter.

VF testing was conducted using either a Perimeter Octopus 101 or Octopus 900 instrument (Haag-Streit AG, Koeniz, Switzerland). The TOP strategy with a G pattern, consisting of 59 points within a central circle of 30°, was used for all VF tests included in this analysis. The parameters and examination protocols of the perimeter were as previously described [19]. Results from tests with reliability factor scores greater than 25% were excluded from the analysis to avoid significant positive or negative bias. The mean defect (MD), also a standard output of Octopus, was used to determine the extent of VF defects. As VF testing might not be a reliable measure of visual function in the pediatric population [20], we excluded the data of carriers younger than 14 years.

The ffERG (RetiPort ERG system; Roland Consult, Wiesbaden, Germany) was performed using corneal “ERG jet” contact lens electrodes, according to the standards published by the International Society for Clinical Electrophysiology of Vision. In this study, the b-wave amplitudes of scotopic 3.0 electroretinogram (ERG) and photopic 3.0 ERG responses were selected for ffERG analysis.

### Novel grading system of fundus phenotype

**Fig. 2:**
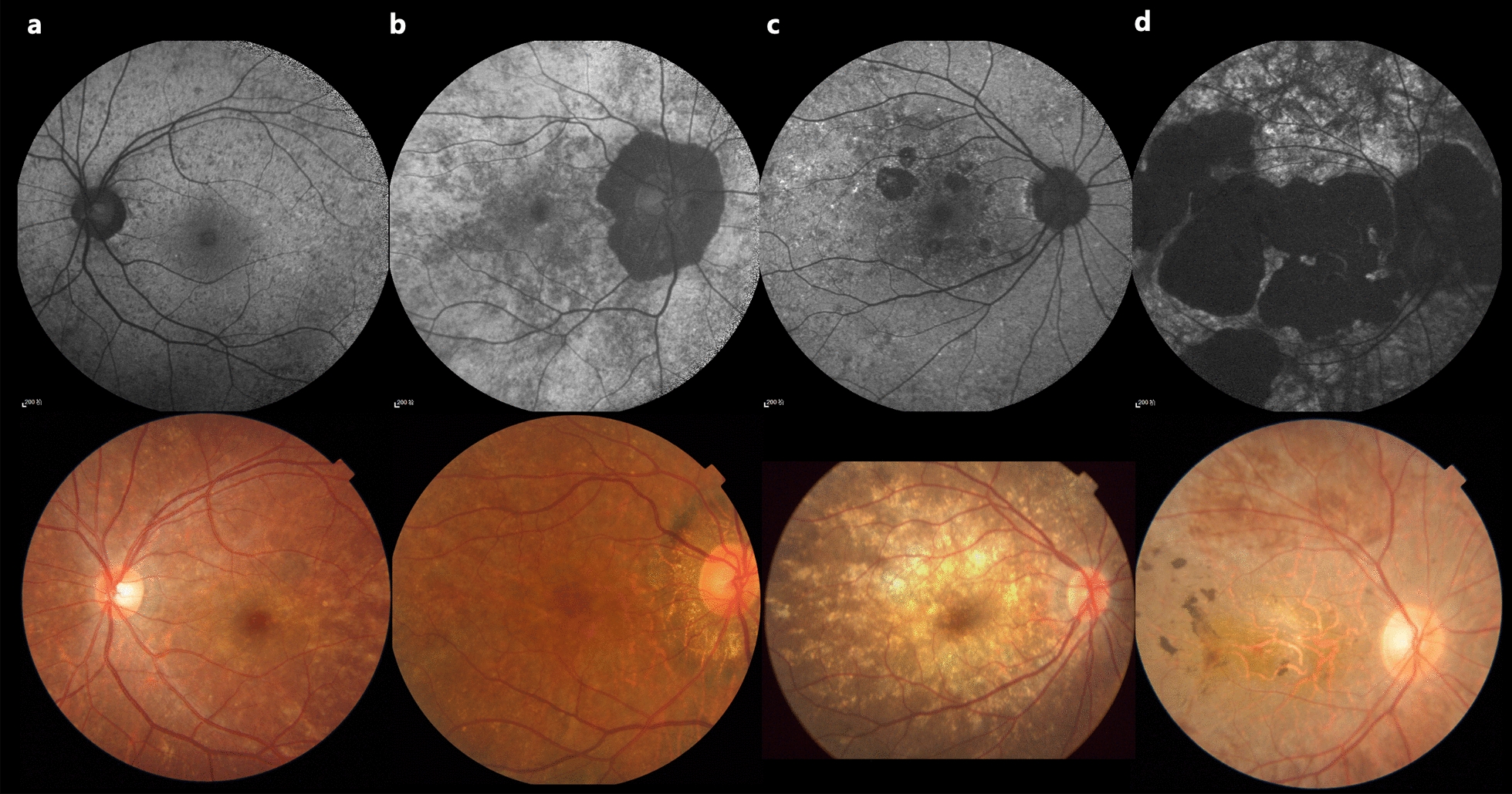
Novel grading system of fundus phenotypes of female carriers with choroideremia based on fundus autofluorescence and fundus color photographs. **a** Granular type; **b** Severe peripapillary atrophy type; **c** Localized atrophy type; **d** Widespread atrophy type

Based on 55° FAF and fundus color photographs, we proposed a new grading system with four fundus phenotypes (Fig. 2):

### Granular type

Fine or coarse areas of hypoautofluorescence without chorioretinal atrophy or with mild peripapillary chorioretinal atrophy that do not meet the criteria for severe peripapillary atrophy type.

### Severe peripapillary atrophy type

Presence of severe peripapillary atrophy, defined as chorioretinal atrophy encompassing the optic disc, with a horizontal atrophy width at least twice the horizontal disc diameter, in the absence of other chorioretinal atrophy beyond the peripapillary atrophic lesion.

### Localized atrophy type

Presence of focal chorioretinal atrophy located beyond the peripapillary atrophic lesion, with a total atrophy area of less than five-disc areas, including peripapillary atrophy.

### Widespread atrophy type

Presence of widespread chorioretinal atrophy, with a total atrophy area of at least five-disc areas, including peripapillary atrophy.

Table 1 shows the differences between the two existing grading systems and the system proposed in the current study.

**Table 1:** Summary of the three grading systems for female carriers of choroideremia

Edwards et al. [15]	Jauregui et al. [16]	Current study
Fine Coarse Geographic Male pattern	Mild: Defined as presenting without areas of chorioretinal atrophy Intermediate: Defined as presenting with a smaller, localized area of chorioretinal atrophy Severe: Defined as presenting with widespread chorioretinal atrophy throughout the posterior pole	Granular: Granular-like hypoautofluorescence without chorioretinal atrophy or with mild peripapillary chorioretinal atrophy Severe peripapillary atrophy: Presence of severe peripapillary atrophy, defined by horizontal atrophy width at least twice the horizontal disc diameter Localized atrophy: Presence of chorioretinal atrophy beyond the peripapillary atrophic lesion, and the total atrophy area is less than five-disc areas Widespread atrophy: The total atrophy area of at least five-disc areas

### Agreement assessment on grading

To assess agreement on gradings, two distinct grading methods were employed: measurement-based grading and visual assessment grading. For measurement-based grading, an IRD specialist (XH) manually measured (i) the horizontal width of peripapillary atrophy and the horizontal disc diameter for all cases suspected as severe peripapillary atrophy and (ii) the total atrophy area and disc area for cases suspected as localized or widespread atrophy. These measurements were then reviewed by a second IRD specialist (RS). For any disputed cases, the two ophthalmologists referred to fundus color photographs, OCT, and infrared reflectance images to reach a consensus on the boundaries of atrophy or the optic disc. After finalizing the measurements, all eyes were graded according to the proposed grading system based on fundus phenotype and measurement data, and these established gradings were used for subsequent data analysis. For visual assessment grading, a third IRD specialist (XZ), blinded to the measurement results, performed gradings based solely on visual inspection of fundus color photographs and FAF images. Agreement between the visual assessment and the measurement-based grading was analyzed using Cohen’s Kappa coefficient.

### Genetic testing

Genomic DNA was extracted from peripheral blood samples for genetic testing with a QIAamp DNA Blood Midi Kit (Qiagen, Hilden, Germany) according to the manufacturer’s instructions. In families with genetically confirmed disease-causing variants identified in male probands, obligate carriers underwent validation using Sanger sequencing or multiplex ligation-dependent probe amplification (MLPA). MLPA was performed with SALSA MLPA probe mix P366-A2 CHM-RP2-RPGR (Lot A2-0614; MRC-Holland, Amsterdam, the Netherlands) according to the manufacturer’s instructions. In families lacking established disease-causing variants, carriers were genetically confirmed using next-generation sequencing panels or whole-exome sequencing.

### Statistical analysis

For descriptive analysis, continuous variables are presented as means ± standard deviations (SDs) or medians and quartiles, as appropriate. We used the intraclass correlation coefficient (ICC) method to assess interocular symmetry at baseline (first visit) for visual function parameters and the Cohen’s kappa coefficient to assess both interocular symmetry of the fundus phenotype and agreement between gradings. To account for repeated measurements from both eyes of each patient, we applied a mixed-effects linear regression model with random intercept and slope to estimate progression rates by age. To investigate potential differences in age, visual parameters, and genotype across phenotypic subgroups, we assessed correlations between these metrics and retinal phenotype grading using either the Kruskal–Wallis test or one-way ANOVA, selected based on variance homogeneity and normality. Upon identifying significant intergroup differences (*P* < 0.05), post-hoc pairwise comparisons were performed using the Dunn’s test to control for type I error inflation, and adjusted *P* values were reported for all multiple comparisons, with statistical significance maintained at α = 0.05. Baseline and follow-up data were included for all analyses except for interocular symmetry and genotype-retinal phenotype correlation. The analyses were performed using SPSS (version 27.0, IBM Corp., Armonk, NY, USA) and GraphPad Prism (GraphPad Software, Inc., La Jolla, CA, USA). Statistical significance was set at a two-sided *P* < 0.05.

## Results

### Study participants

This study included 128 eyes of 64 female carriers of *CHM* mutations, all of Han Chinese ethnicity, and represented 46 distinct families. The enrolled carriers comprised (i) symptomatic individuals seeking medical evaluation (5/64 carriers, 7.8%), (ii) asymptomatic relatives self-referring for ophthalmic evaluation owing to affected family members (10/64 carriers, 15.6%), (iii) asymptomatic individuals diagnosed incidentally during routine examinations (1/64 carriers, 1.6%), and (iv) asymptomatic relatives invited for evaluation based on familial affected males (48/64 carriers, 75.0%). The genotypic and phenotypic characteristics of the carriers are summarized in Table 2. The fundus phenotypes and visual functional profiles of the carriers at baseline are presented in Table 3. Follow-up data were collected for 26 eyes from 13 carriers. Follow-up visits with an interval of ≥ 1 year from the preceding visit were included, yielding a total of 35 longitudinal data points (including baseline visits) from these 13 carriers. The median number of visits was 2 [interquartile range (IQR) 2–3] with a median follow-up period of 4.4 years (IQR 2.3–10.4 years). Among the 64 carriers, two carriers presented with night blindness, while five presented with decreased vision.

**Table 2:** The genotypic and phenotypic characteristics of choroideremia carriers

Family ID	Sequence variant^a^		Patient ID	Age	Fundus phenotype		BCVA/logMAR		Scotopic 3.0 ERG/μV		Photopic 3.0 ERG/μV			VF-MD/dB	
					OD	OS	OD	OS	OD	OS		OD	OS	OD	OS
1	c. 315-1G > C		1	58.7	Granular	Localized	0.0000	0.0000	565	551		168	148	NA	NA
				63.8	Granular	Localized	0.0000	0.0000	NA	NA		NA	NA	6.1	4.6
2	c. 525_526delAG	p. Glu177Lysfs*6	2	36.3	Granular	Granular	0.3010	− 0.1761	638	579		227	217	− 1.2	− 2.1
				39.2	NA	NA	0.3010	− 0.0792	NA	NA		NA	NA	NA	NA
				41.5	NA	NA	0.3010	− 0.0792	NA	NA		NA	NA	NA	NA
				42.5	NA	NA	0.3010	− 0.0792	NA	NA		NA	NA	NA	NA
				50.5	Granular	Granular	0.3979	0.0000	NA	NA		NA	NA	3.3	0.7
			3	59.7	Granular	Granular	0.0969	0.0969	NA	NA		NA	NA	NA	NA
				62.0	Granular	Granular	0.0969	0.0969	NA	NA		NA	NA	NA	NA
3	c. 1342C > T	p. Gln448*	4	44.3	NA	NA	0.0000	0.0000	NA	NA		NA	NA	NA	NA
4	c. 1565C > A	p. Ser522*	5	54.1	Granular	Granular	0.5229	0.5229	NA	NA		NA	NA	NA	NA
5	c. 877C > T	p. Arg293*	6	21.3	Granular	Granular	0.0000	0.0000	NA	NA		NA	NA	NA	NA
6	c. 703-2A > G		7	44.3	Granular	Granular	0.0000	0.0000	NA	NA		NA	NA	NA	NA
			8	20.8	Granular	Granular	0.0000	0.0000	NA	NA		NA	NA	NA	NA
7	c. 50-1G > A		9	26.4	NA	NA	0.0000	0.0000	548	531		116	134	− 0.4	− 0.3
8	c. 116 + 1G > A		10	59.0	NA	NA	0.2218	0.0969	NA	NA		NA	NA	NA	NA
			11	50.0	NA	NA	0.0969	0.0969	NA	NA		NA	NA	NA	NA
			12	47.0	NA	NA	0.0000	0.0000	NA	NA		NA	NA	NA	NA
9	E1-E15 deletion		13	29.8	Granular	Granular	− 0.1761	− 0.1761	NA	NA		NA	NA	NA	NA
				44.4	Granular	Granular	− 0.0792	− 0.0792	NA	NA		NA	NA	1.2	0.8
			14	63.2	NA	NA	0.2218	0.3010	NA	NA		NA	NA	NA	NA
10	c. 1130 T > A	p. Leu377*	15	71.0	NA	NA	0.3010	0.2218	NA	NA		NA	NA	NA	NA
			16	67.1	NA	NA	0.5229	0.2218	NA	NA		NA	NA	NA	NA
11	c. 612_613delAG	p. Glu204fsX222	17	69.0	Localized	Localized	0.5229	0.2218	247	309		75	88	NA	NA
			18	17.3	NA	NA	0.0000	0.0000	NA	NA		NA	NA	NA	NA
12	c. 189 + 2 T > C		19	34.1	Granular	Granular	− 0.0792	− 0.1761	NA	NA		NA	NA	NA	NA
				46.1	Granular	Granular	0.0000	0.0000	NA	NA		NA	NA	6.6	9.2
			20	47.5	NA	NA	0.0969	0.2218	NA	NA		NA	NA	NA	NA
			21	50.5	NA	NA	0.0000	0.0000	NA	NA		NA	NA	NA	NA
			22	42.5	NA	NA	0.2218	0.0969	NA	NA		NA	NA	NA	NA
13	c. 315-2_316delAGTC		23	43.1	Granular	Granular	0.0000	0.0969	NA	NA		NA	NA	NA	NA
14	c. 140G > A	p. Trp47*	24	50.2	Granular	Granular	0.0000	0.0000	646	645		190	158	3.3	5.8
15	c. 1349 + 2 T > C		25	49.0	Granular	Granular	0.0000	0.0969	NA	NA		NA	NA	3.1	3.6
16	c. 543delT	p. Cys182fsX198	26	25.5	Granular	Granular	0.0969	0.0000	NA	NA		NA	NA	1.4	0
17	E3-E15 deletion		27	54.1	Granular	Granular	0.0000	0.0000	NA	NA		NA	NA	6.3	7.7
			28	3.2	Granular	NA	0.3979	0.5229	NA	NA		NA	NA	NA	NA
18	c. 49 + 5G > C		29	80.3	Localized	Localized	0.3010	0.6990	NA	NA		NA	NA	NA	NA
19	E4 duplication		30	64.0	NA	NA	0.0458	0.0458	NA	NA		NA	NA	NA	NA
			31	14.6	Granular	Granular	0.0000	0.0000	NA	NA		NA	NA	1.6	3.2
20	c. 715C > T	p. Arg239*	32	32.5	Widespread	Peripapillary	0.0969	0.0000	228	482		92	167	10	1.5
				33.6	NA	NA	0.0969	0.0000	NA	NA		NA	NA	10.5	1.4
				39.0	Widespread	Peripapillary	0.3010	0.0000	NA	NA		NA	NA	NA	NA
				42.9	Widespread	Peripapillary	0.6021	0.0969	124	353		41	77	15.2	2.1
21	c. 190-2A > C		33	50.2	NA	NA	0.0000	0.0000	366	293		23	24	NA	NA
22	c. 315-1G > C		34	55.6	Granular	Granular	0.0000	0.0000	624	510		129	169	NA	NA
23	c. 808C > T	p. Arg270*	35	49.5	Granular	Granular	0.0000	0.0000	NA	NA		NA	NA	NA	NA
24	c.757C > T	p. Arg253*	36	23.6	Granular	Granular	− 0.0792	− 0.0792	NA	NA		NA	NA	3.7	1.4
25	c.1370 T > C	p. Leu457Pro	37	66.7	Localized	Peripapillary	− 0.0792	0.2218	NA	NA		NA	NA	NA	NA
26	E2 deletion		38	61.2	Peripapillary	Peripapillary	0.0969	0.0969	NA	NA		NA	NA	6.8	4.5
27	c.1350-11_c.1350-10insACAGGCAGTAAAAGGCAGTTATA		39	25.0	Granular	Granular	0.0000	0.0000	549	549		141	133	1.9	2
				28.1	Granular	Granular	0.0000	0.0000	NA	NA		NA	NA	NA	NA
28	c.1584_1587delTGTT	p. Val529Hisfs*7	40	71.7	NA	NA	0.3010	0.2218	NA	NA		NA	NA	NA	NA
29	E7 deletion		41	28.8	Localized	Widespread	0.0969	0.0969	NA	NA		NA	NA	7.2	10
				31.3	Localized	Widespread	0.0000	0.0969	224	171		116	64	6.1	15.6
				32.9	NA	NA	0.0969	0.0969	NA	NA		NA	NA	9.8	14.6
				34.2	Widespread	Widespread	0.0969	0.0969	153	73		94	49	11.5	17.8
30	c.545delG	p. Cys182fs	42	44.9	Granular	Granular	− 0.0792	− 0.0792	NA	NA		NA	NA	NA	NA
				49.0	Granular	Granular	0.0000	0.0000	NA	NA		NA	NA	2.9	2.9
			43	42.7	NA	NA	− 0.0792	− 0.0792	NA	NA		NA	NA	NA	NA
			44	43.9	Granular	Granular	0.0969	0.0969	NA	NA		NA	NA	2.5	3.1
31	E2 deletion		45	41.5	Granular	Granular	0.3010	0.0969	NA	NA		NA	NA	NA	NA
32	E3-E4 deletion		46	39.9	Localized	Granular	0.0969	0.0000	504	511		168	163	0.8	0.3
				40.9	Localized	Granular	0.0969	0.0000	NA	NA		NA	NA	− 0.3	0.3
				41.9	Localized	Granular	0.0969	0.0000	NA	NA		NA	NA	NA	NA
33	c.525_526delAG	p. Glu177Lysfs*6	47	4.9	NA	NA	0.0969	0.3010	NA	NA		NA	NA	NA	NA
34	c.757C > T	p. Arg253*	48	46.5	Granular	Peripapillary	0.0000	0.0000	NA	NA		NA	NA	2.4	1.9
			49	26.7	Granular	Granular	0.0000	0.0000	NA	NA		NA	NA	1.1	3.3
35	c.616dupA	p. Thr206Asnfs*17	50	36.5	Granular	Peripapillary	0.0000	0.2218	NA	NA		NA	NA	0.7	2.1
36	c.877C > T	p. Arg293*	51	51.5	Localized	Widespread	− 0.0792	0.0000	NA	NA		NA	NA	NA	NA
				53.7	Localized	Widespread	0.0000	0.0969	459	487		185	160	3.5	6.8
				55.8	Widespread	Widespread	0.0000	0.2218	430	411		141	128	1.8	7
37	c.1771-1G > A		52	10.6	Granular	Granular	− 0.0792	− 0.0792	NA	NA		NA	NA	NA	NA
			53	8.4	Granular	Granular	0.0000	0.0000	NA	NA		NA	NA	NA	NA
38	E1-E15 deletion		54	27.1	Granular	Granular	0.0000	0.0000	NA	NA		NA	NA	6.4	4.1
39	E9-E15 deletion		55	36.9	Granular	Localized	0.0000	0.0000	NA	NA		NA	NA	1.2	1.2
40	c.715C > T	p. Arg239*	56	44.6	Granular	Peripapillary	0.0000	0.0000	NA	NA		NA	NA	1.2	2.8
41	c.808C > T	p. Arg270*	57	6.9	Granular	Granular	0.0969	0.0458	NA	NA		NA	NA	NA	NA
42	c.1585delG	p. Val529Phefs*8	58	29.7	Widespread	Widespread	0.0000	0.0000	NA	NA		NA	NA	21.2	22.3
43	c.1584_1587delTGTT	p. Val529Hisfs*7	59	44.6	Granular	Granular	− 0.0792	− 0.0792	NA	NA		NA	NA	− 0.1	1.8
44	c.355C > T	p. Gln119*	60	34.7	Granular	Granular	0.0000	0.0000	NA	NA		NA	NA	NA	NA
				36.4	Granular	Granular	0.0000	0.0000	517	507		158	142	2.3	0.9
			61	14.1	Granular	Granular	0.0000	0.0000	NA	NA		NA	NA	NA	NA
				15.5	Granular	Granular	0.0000	0.0000	NA	NA		NA	NA	2.9	3.2
45	c.1530C > A	p. Cys510*	62	31.8	Granular	Granular	0.0000	0.0000	NA	NA		NA	NA	− 0.3	0.1
46	E2 deletion		63	13.7	Granular	Granular	− 0.0792	− 0.0792	NA	NA		NA	NA	NA	NA
			64	56.2	Granular	Granular	0.0000	0.0000	NA	NA		NA	NA	4	5.3

^**a**^Reference NM_000390.4

*BCVA* = best-corrected visual acuity; *Scotopic 3.0 ERG* = b-wave amplitude of scotopic 3.0 electroretinography; *Photopic 3.0 ERG* = b-wave amplitude of photopic 3.0 electroretinography; *VF-MD* = mean defect values of visual field; *NA* = not available

**Table 3:** Baseline characteristics of choroideremia carriers

Baseline characteristics	
Age (years)	Data from 64 carriers
Mean ± SD	40.7 ± 18.2
Fundus phenotype	Data from 93 eyes
Granular	76.3% (71/93 eyes)
Severe peripapillary atrophy	7.5% (7/93 eyes)
Localized atrophy	10.8% (10/93 eyes)
Widespread atrophy	5.4% (5/93 eyes)
Visual function^a^	
BCVA (logMAR)	Data from 128 eyes
Median (IQR)	0.000 (0.000–0.097)
VF-MD (dB)	Data from 60 eyes
Median (IQR)	2.9 (1.2–5.1)
3.0 scotopic ERG (μV)	Data from 28 eyes
Median (IQR)	508.5 (297.0–550.5)
3.0 photopic ERG (μV)	Data from 28 eyes
Median (IQR)	141.5 (89.0–167.8)

^a^Analysis is based on binocular visual function data

*BCVA* = best-corrected visual acuity; *VF-MD* = mean defect values of visual field; *3.0 scotopic ERG* = b-wave amplitude of scotopic 3.0 electroretinography; *3.0 photopic ERG* = b-wave amplitude of photopic 3.0 electroretinography; *IQR* = interquartile range; *SD* = standard deviation

### Agreement on grading

A total of 127 eyes from 64 follow-up visits were evaluated using both measurement-based grading and visual assessment grading. The results showed discrepancies in only five eyes between the two grading methods: four eyes graded as widespread atrophy by measurement were graded as localized atrophy by visual assessment, and one eye graded as severe peripapillary atrophy by measurement was graded as granular type by visual assessment. Statistical analysis showed a weighted κ of 0.93 [95% confidence interval (CI): 0.89–0.99], indicating excellent agreement between the two methods. This demonstrates that visual assessment alone can achieve high agreement with measurement-based grading.

### Interocular symmetry

The interocular symmetry at baseline, assessed using ICC, was moderate for BCVA (ICCs = 0.691) and high for the MD values of VF (ICCs = 0.876), scotopic ERG (ICCs = 0.891), and photopic ERG (ICCs = 0.862). The interocular symmetry for fundus grading was moderate, with κ = 0.46 (95% CI: 0.24–0.69, *P* < 0.001) assessed using the kappa coefficient.

### Retinal features

At baseline, yellowish spots or streaks in the fundus were the most common manifestation, observed in 109 of 128 eyes (85%) on the color fundus photographs (Fig. 2). Peripapillary atrophy was the second most frequent manifestation, observed in 101 of 128 eyes (79%).

Overall, 93 eyes underwent FAF examination at baseline. Mosaic patches of relative hyperautofluorescence and hypoautofluorescence were the most common FAF manifestation.

### Fundus grading at baseline

Measurement-based grading results were used for all subsequent descriptions and analyses. Among the cohort, 93 eyes from 47 CHM carriers with available baseline color fundus photographs and FAF images were included for baseline grading. Thirty-five eyes from 18 carriers could not be graded due to the lack of FAF results. Among the 93 gradable eyes, 71 (76.3%) were of the granular type, seven (7.5%) were of the severe peripapillary atrophy type, 10 (10.8%) were of the localized atrophy type, and five (5.4%) were of the widespread atrophy type. Notably, of the 10 eyes with localized atrophy, seven (70.0%) showed severe peripapillary atrophy. Among the five eyes with widespread atrophy, four (80.0%) also exhibited severe peripapillary atrophy.

Both localized and widespread chorioretinal atrophy in the carriers were mainly located in the posterior pole and peripapillary regions (Fig. 2c and d). This is inconsistent with the pattern observed in male patients, where atrophy typically originates in the mid-peripheral retina and progresses toward the posterior pole.

### Longitudinal changes in fundus grading and area of retinal atrophy

**Fig. 3:**
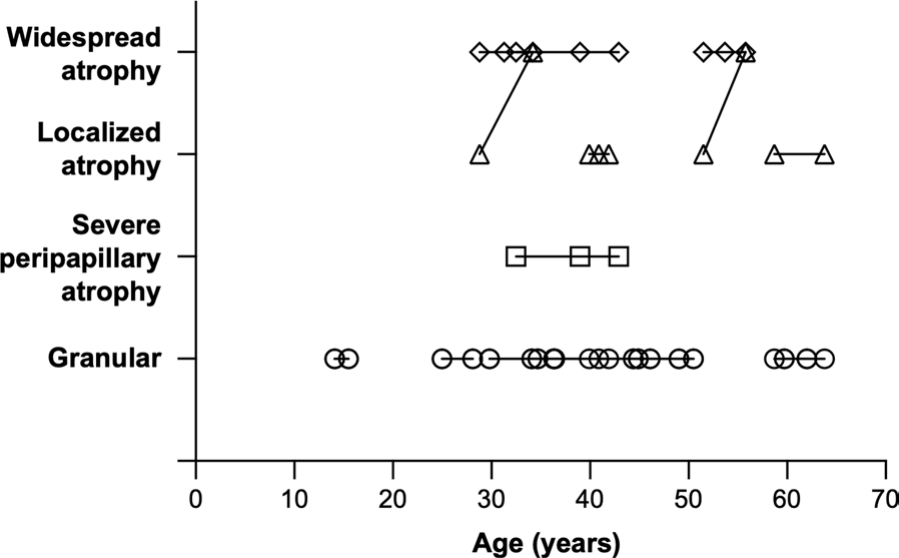
Longitudinal changes in fundus grading. Longitudinal changes in fundus grading, showing 26 eyes from 13 carriers with a median follow-up period of 4.4 years (IQR 2.3–10.4 years). Circular, square, triangular, and diamond symbols represent eyes with baseline gradings of granular, severe peripapillary atrophy, localized atrophy, and widespread atrophy type, respectively

**Fig. 4:**
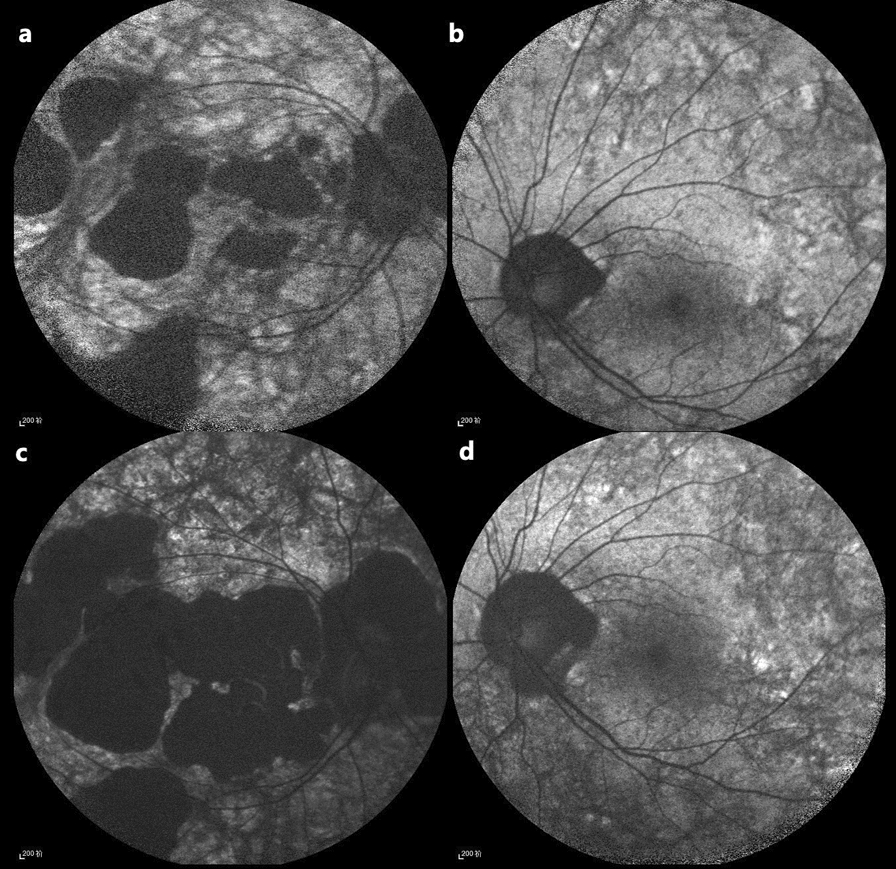
Disease progression in a choroideremia carrier. **a**, **b** Fundus autofluorescence (FAF) images of the right and left eyes, respectively, at baseline (age 32.5 years). **c**, **d** Corresponding FAF images at final follow-up (age 42.9 years). Although the fundus grading remained unchanged between baseline and final follow-up (right eye: widespread atrophy; left eye: severe peripapillary atrophy), an enlargement of the hypo-autofluorescent areas was observed bilaterally, indicating disease progression

Overall, 26 eyes of 13 CHM carriers had longitudinal follow-up data on fundus grading; longitudinal grading changes are illustrated in Fig. 3. Among the 18 eyes with the granular type at baseline, all eyes remained granular after a median follow-up of 3.6 years (IQR 2.1–12.0 years), indicating no progression to severe peripapillary or localized atrophy, regardless of the granule size (fine or coarse). This further supports the lack of clinical significance in distinguishing between fine and coarse granular subtypes and indicates a relatively good prognosis for the granular type. One eye with severe peripapillary atrophy type at baseline remained in the severe peripapillary atrophy type after 10.4 years of follow-up; however, the area of peripapillary retinal atrophy expanded (Fig. 4b and d). Among the four eyes with localized atrophy at baseline, two eyes (50%) progressed to the widespread atrophy type after a median follow-up of 3.6 years (IQR 3.8–5.2 years). All four eyes exhibited expansion of the retinal atrophic area. All three eyes with widespread atrophy at baseline exhibited an expansion of atrophy areas after a median follow-up of 5.3 years (IQR 4.9–7.9 years) (Fig. 4a and c). These results suggest that the conditions of all non-granular types progress, manifesting as an enlargement of retinal atrophy areas over time.

As we found that the areas of retinal atrophy in the eyes with severe peripapillary, localized, and widespread atrophy at baseline expanded during follow-up, we measured the annual progression rates of the atrophy areas in these eight eyes. The mean annual progression rate of these eight eyes was 3.1 mm^2^/year, calculated as the mean annual progression rate of each eye. Furthermore, we calculated the annual retinal atrophy progression rates for these three subtypes separately. The mean progression rates for the severe peripapillary, localized and widespread atrophy types were 0.32, 2.13, and 5.28 mm^2^/year, respectively, indicating distinct prognoses across the subtypes, particularly a graded increase in severity.

### Analysis of visual function progression with age

Age-related progression rates of visual function parameters were calculated based on data from the entire cohort using two methods: mixed-effects linear regression for binocular data and linear regression for worse-eye data. The results are presented in Table 4. These two analyses yielded relatively consistent results: BCVA exhibited a minimal and clinically insignificant deterioration trend with increasing age. No significant linear progression with age was observed for VF and ERG responses.

**Table 4:** Visual function progression with age

Visual function parameter		Progression rate using mixed-model linear regression based on binocular data				Progression rate using linear regression based on worse-eye data			
	Data source	Mean	SE	*P* value	R^2^	Mean	SE	*P* value	R^2^
BCVA (logMAR)	86 clinical visits from 64 carriers	0.003	0.001	< 0.001	0.084	0.003	0.001	0.002	0.108
VF-MD (dB)	40 clinical visits from 31 carriers	− 0.01	0.05	0.85	0.001	− 0.01	0.08	0.88	0.001
3.0 scotopic ERG (μV)	17 clinical visits from 14 carriers	2.50	2.58	0.34	0.029	2.56	3.90	0.52	0.028
3.0 photopic ERG (μV)	17 clinical visits from 14 carriers	0.41	0.80	0.61	0.008	0.52	1.14	0.65	0.014

*BCVA* = best-corrected visual acuity; *SE* = standard error; *VF-MD* = mean defect values of visual field; *3.0 scotopic ERG* = b-wave amplitude of scotopic 3.0 electroretinography; *3.0 photopic ERG* = b-wave amplitude of photopic 3.0 electroretinography

### Analysis of fundus phenotype–age correlation

**Fig. 5:**
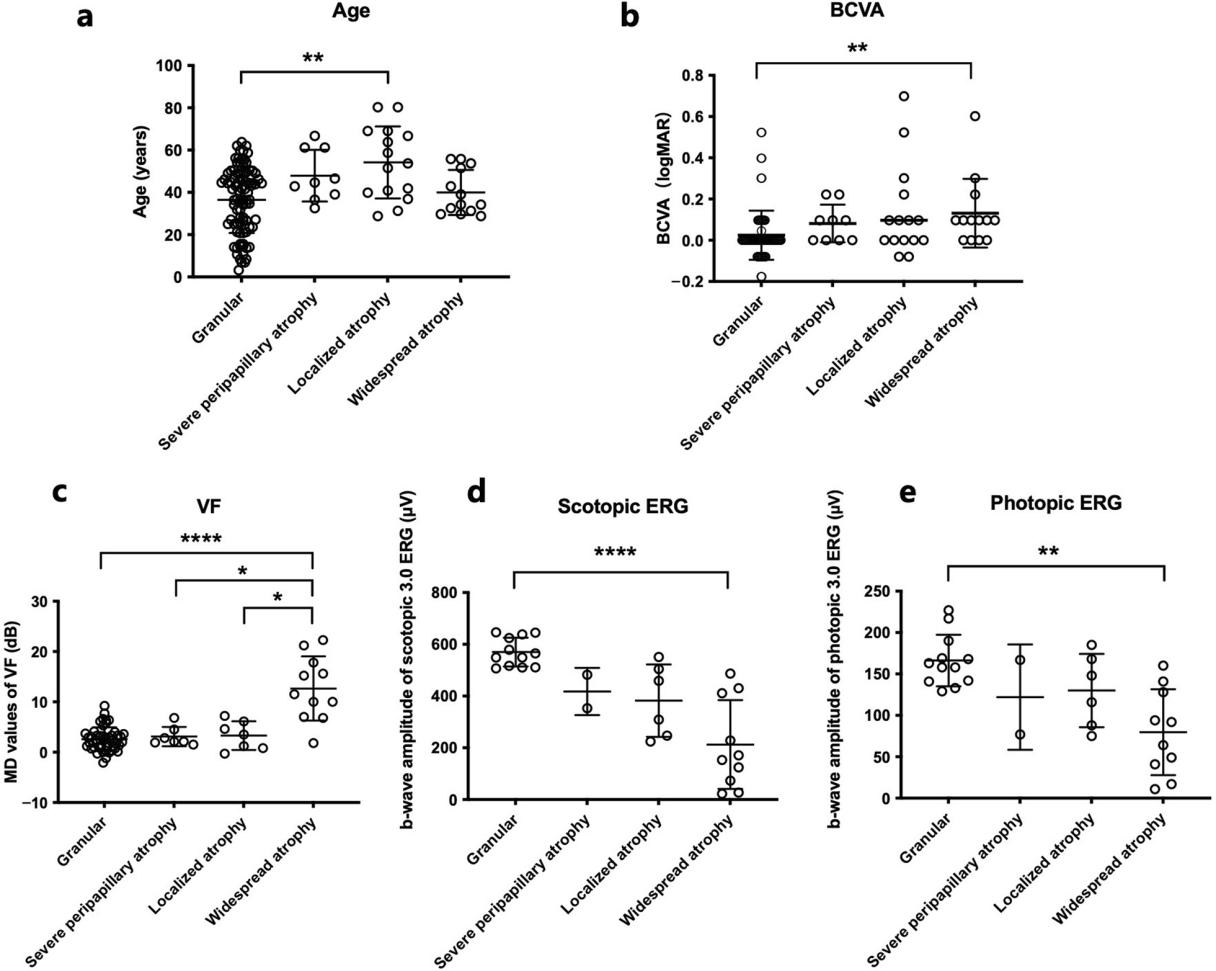
Differences in age and functional parameters among fundus phenotypic subtypes of choroideremia carriers. **a** Age distribution; **b** Best-corrected visual acuity (BCVA, logMAR); **c** Mean defect values of the visual field (dB); **d** b-wave amplitude of scotopic 3.0 electroretinogram (ERG; μV); **e** b-wave amplitude of photopic 3.0 ERG (μV). The lines represent the mean and standard deviation. *P < 0.05; **P < 0.01; ****P < 0.0001. MD, mean defect; VF, visual field

To examine the correlation between age and fundus phenotype grading, we conducted Kruskal*–*Wallis tests, which revealed a significant age difference between at least two grading groups (H = 12.85, df = 3, *P* = 0.005). Post-hoc Dunn’s test indicated that carriers with localized atrophy were significantly older (adjusted *P* = 0.007) than those with the granular type (Fig. 5). These results demonstrated that overall severity does not exhibit an increase with age.

### Analysis of fundus phenotype–visual function correlation

The Kruskal–Wallis test revealed significant differences among the four phenotypic groups in BCVA (H = 17.85, df = 3, *P* < 0.001), MD values of VF (H = 21.42, df = 3, *P* < 0.0001), and b-wave amplitudes of scotopic (H = 20.99, df = 3, *P* = 0.0001) and photopic 3.0 ERG (H = 12.38, df = 3, *P* = 0.006). Post-hoc Dunn’s tests revealed that the widespread atrophy type had significantly worse BCVA (adjusted *P* = 0.004), and lower b-wave amplitudes of scotopic (adjusted *P* < 0.0001) and photopic ERG (adjusted *P* = 0.003), compared to the granular type (Fig. 5). Furthermore, the widespread atrophy type showed significantly more severe VF loss than the granular (adjusted *P* < 0.0001), severe peripapillary atrophy (adjusted *P* = 0.044), and localized atrophy types (adjusted *P* = 0.035) (Fig. 5).

### Analysis of fundus phenotype-genotype correlation

Genetic test results confirmed the clinical diagnosis of CHM carriers for all participants. The list of documented sequence variants and their predicted effects is provided in Table 1. Overall, 36 different sequence variants were detected in 46 families, including 11 splicing sequence variants, 10 nonsense sequence variants, seven copy number variations, six small deletion sequence variants, one small duplication sequence variant, and one missense sequence variant.

To explore the correlation between genotypes and fundus phenotypes, nonsense, small deletions, and small duplications were combined into a single group. This group, along with the splicing, copy number, and missense variant groups, constituted the four genotype groups used to analyze correlations with the fundus phenotypes. The Kruskal–Wallis test showed no significant differences in the genotype distribution among the fundus phenotypes (H = 6.34, df = 3, *P* = 0.096). Given the potential effect of a single missense variant, we performed a subsequent analysis excluding this group. The correlation between the remaining three genotype groups and fundus phenotypes remained non-significant (H = 1.12, df = 2,* P* = 0.570), and thus yielded no evidence for supporting a genotype–phenotype correlation.

## Discussion

Owing to skewed X-chromosome inactivation and other potential factors, CHM carriers display marked variability in clinical manifestations, with a minority developing severe manifestations that may necessitate therapeutic intervention. Therefore, a comprehensive understanding of the natural history, proportion of severe cases, the indicators predicting the disease trajectory, and the interocular symmetry of these female carriers is essential for clinical counseling, treatment plan selection, and identifying candidates for future interventions. This study aims to contribute to the existing knowledge on these aspects using a large cohort of CHM carriers. In addition, this study proposes an improved grading system to better distinguish carrier disease conditions and prognoses, thereby guiding clinical treatment.

Using data from 43 CHM carriers and employing Edwards’ four-tier grading system, Gocuk et al. [13] reported significant differences in low-luminance visual acuity (LLVA) (*P* = 0.04), but not in BCVA, low luminance deficit, and Macular Integrity Assessment microperimetry parameters between the fine and coarse types. Edwards et al. [15] also reported no significant differences in retinal threshold sensitivity between the fine and coarse types. These findings suggest that visual function may not differ significantly between the two types. Moreover, distinguishing between fine and coarse types poses challenges in clinical practice due to the absence of clear criteria. Considering these, we merged the two types into a single category in our revised fundus grading system. Parmann et al. [21] identified peripapillary atrophy in 14 of 18 CHM carrier eyes (77.8%), suggesting this feature is a characteristic manifestation among CHM carriers. In this study, we established specific criteria for severe peripapillary atrophy. Subsequent observations revealed that 70% of eyes with localized atrophy and 80% with widespread atrophy exhibited severe peripapillary atrophy, with progression in the atrophy area during follow-up. These findings led us to hypothesize that severe peripapillary atrophy may represent an early or essential manifestation of localized or widespread atrophy and may potentially indicate progression. Consequently, we designated severe peripapillary atrophy as a distinct subtype to emphasize its clinical significance in disease assessment and prognostic prediction. Considering these aspects, we propose a novel four-tier fundus grading system, enhanced by clear definitions to improve grading accuracy and reproducibility. We demonstrated high agreement between visual assessment and measurement-based grading, supporting the reliability of visual assessment alone for clinical application while maintaining grading accuracy.

According to our grading system, the granular type was predominant (76.3%), followed by localized atrophy (10.8%), severe peripapillary atrophy (7.5%), and widespread atrophy (5.4%) types, indicating predominantly mild fundus manifestations in CHM carriers. This is consistent with Gocuk’s report of combined fine/coarse types, accounting for 68% of the eyes [13]. However, as approximately 75% of carriers in this study were asymptomatic relatives invited owing to having affected family members, this may have caused an overestimation of the proportion of mild phenotypes. Further large-sample studies are therefore warranted.

Our longitudinal results showed that all granular-type eyes at baseline exhibited no progression in fundus grading, regardless of granule size (fine or coarse). This implies that differentiation between fine and coarse granules has little clinical significance, suggesting that the granular type is associated with a favorable prognosis. This is consistent with the findings of Gocuk et al., who reported that neither the fine nor the coarse types progressed to geographic atrophy [17]. Conversely, all eyes with severe peripapillary, localized, and widespread atrophy types showed a progressive expansion of retinal atrophy, with each type demonstrating a more rapid progression, indicating a poorer prognosis for these three types. Therefore, baseline fundus grading is a critical prognostic indicator of disease outcome. In addition, our analyses revealed a slow and clinically negligible decline in VA with advancing age. Neither VF parameters nor ffERG amplitudes demonstrated significant age-related deterioration. These findings indicate that age is not a clinically significant risk factor for functional visual decline among the entire cohort. Our results also showed that neither advancing age nor genotype was associated with phenotype severity. Taken together, these demonstrate that baseline fundus grading is a superior prognostic indicator compared with other parameters, enabling the clinical prediction of disease prognosis at the initial visit.

Our results showed that the progression rates of retinal atrophy for the severe peripapillary atrophy, localized atrophy, and widespread atrophy types were 0.32, 2.13, and 5.28 mm^2^/year, respectively, which are consistent with the progression rates reported by Gocuk et al. [17] for female carriers with severe phenotypes (geographic atrophy: 2.5 mm^2^/year, male pattern: 3.7 mm^2^/year). The progression rates, although lower than those observed in male patients [22], remain non-negligible, particularly in the localized and widespread atrophy types, suggesting that carriers with severe phenotypes should undergo more intensive follow-up and may be considered candidates for potential interventional therapies.

Our study revealed moderate interocular symmetry in VA among CHM carriers, which is consistent with previous findings regarding CHM carriers [13] and patients with CHM [23, 24]. Furthermore, the VF and ffERG parameters demonstrated good interocular symmetry, which is consistent with previous studies on patients with CHM [24]. This study observed moderate phenotypic interocular symmetry in fundus grading among CHM carriers. However, the presence of interocular phenotypic discordance in some carriers suggests that therapeutic interventions might be necessary in only one eye for certain individuals. This observed asymmetry likely reflects the broader spectrum of retinal phenotypic variability in carriers, possibly due to one or more mechanisms including skewed X-chromosome inactivation, genomic imprinting, or escape from X-inactivation [14]. A deeper understanding of the precise mechanisms underlying this phenotypic variability could provide valuable insights into the pathogenesis in CHM carriers and enhance our knowledge of other X-linked disorders.

Our study revealed significant differences in BCVA, VF parameters, and ffERG responses between the widespread and granular atrophy types. However, owing to the relatively small sample size, imbalanced distribution among subtypes, and incomplete functional data in some cases, we were unable to conclusively determine whether functional differences exist between other types. This important clinical question warrants further investigation through larger, prospective studies that incorporate additional functional parameters and validation across independent cohorts.

We found that yellowish spots or streaks were the most common findings on color fundus photographs of CHM carriers. Without the assistance of FAF imaging, some carriers may be misdiagnosed with dry age-related macular degeneration owing to the presence of yellowish drusen-like spots in the retina. In female carriers with severe phenotypes, the predominant localization of chorioretinal atrophy in the posterior pole can lead to diagnostic confusion with macular dystrophy. These findings highlight the critical diagnostic considerations that require careful attention and differentiation by ophthalmologists to prevent misdiagnosis and underdiagnosis, particularly in carriers without a definitive family history of CHM.

We highlight some of the study’s limitations. First, owing to its retrospective design, the findings are subject to inherent biases related to participant selection and data collection, and some clinical records were incomplete. Second, longitudinal data were available only for a proportion of carriers, and the follow-up period was relatively short. Third, the clinical assessment parameters were relatively limited; the lack of comprehensive multimodal imaging and functional evaluations, such as OCT, adaptive optics, microperimetry, and LLVA, restricted the depth of structural and functional analyses. Such additional assessments would provide deeper insight into disease mechanisms and assist in validating the proposed grading system. These limitations highlight the need for future prospective longitudinal studies incorporating more sensitive clinical indicators to further validate and extend the present findings.

## Conclusions

We proposed and validated a novel, clinically practical fundus grading system in the largest cohort of CHM carriers with a wide range of disease severities. This system demonstrated strong prognostic value and potential utility in routine monitoring and clinical trial design. Notably, our findings revealed that the phenotypic subtype at baseline, rather than age or genotype, best predicts disease trajectory, highlighting the value of phenotype-based stratification in this population. Our data further suggest that the granular type remains stable over time, whereas the other types show progressive degeneration, with each of these three types showing a progressively faster rate of deterioration. Based on these findings, we recommend that female carriers with these progressive phenotypes receive more intensive follow-up and be considered eligible for future interventions.

## Data Availability

The datasets used in the current study are available from the corresponding author on reasonable request.

## Abbreviations

BCVA: Best-corrected visual acuity
CHM: Choroideremia
CI: Confidence interval
ERG: Electroretinography
FAF: Fundus autofluorescence
ffERG: Full-field electroretinography
ICC: Intraclass correlation coefficient
IQR: Interquartile range
LLVA: Low-luminance visual acuity
logMAR: Logarithm of the minimum angle of resolution
OCT: Optical coherence tomography
VA: Visual acuity
VF: Visual field
MLPA: Multiplex ligation-dependent probe amplification
